# Injection sclerotherapy for the treatment of haemorrhoids in anticoagulated patients

**DOI:** 10.1308/rcsann.2023.0097

**Published:** 2024-01-04

**Authors:** P Batra, A O’Connor, J Walmsley, W Baraza, A Sharma

**Affiliations:** ^1^ Manchester University NHS Foundation Trust, UK; ^2^ The University of Manchester, UK; ^3^ University of Auckland, New Zealand

**Keywords:** Haemorrhoids, Proctology, Injection sclerotherapy, Anorectal

## Abstract

**Background:**

Symptomatic haemorrhoids (SH) are a common condition; however, conventional outpatient treatment, including rubber band ligation, is contraindicated in patients receiving concurrent anticoagulation. Injection sclerotherapy (IST) has been proposed as a treatment option for these patients.

**Methods:**

A retrospective review of case notes was performed in a colorectal surgery department that sits alongside a tertiary cardiothoracic surgical unit. Patients treated with an IST for SH between 1 April 2014 and 30 November 2021 were identified. Anticoagulation was not stopped in these patients as they were at high risk of developing thromboembolism, except in two patients who required alternative procedures. The primary outcome was symptom resolution, defined as no patient reporting bleeding for at least six months. The secondary outcomes were patient-reported complications, number of IST procedures and number of other procedures performed to achieve symptom resolution.

**Results:**

A total of 20 patients with a median age of 64 years (range 35–86, 14 male) who underwent 32 IST treatments were identified. Symptom resolution was achieved in 18 (90%) patients using IST while continuing anticoagulation treatment, with two (10%) patients requiring alternative interventions. Ten patients (50%) required only one IST procedure, and three patients (15%) required two procedures. The remaining five (25%) patients required three or four interventions. The median time between IST treatments was 32 weeks (range 8–133). No complications were reported.

**Conclusion:**

Our study demonstrates that IST can be considered as a potential treatment option for patients with SH who are at a high risk of thromboembolic disease requiring anticoagulation.

## Introduction

Haemorrhoids are common, with a reported prevalence of 13–36%,^1^ and are the most frequent cause of anorectal bleeding.^2^ The Goligher classification system is commonly used to classify haemorrhoids into four grades according to the degree of prolapse (Table 1).^3^ In patients in whom conservative management fails, rubber band ligation (RBL) can be performed in an outpatient setting for grade 1–3 haemorrhoids. However, RBL is contraindicated in anticoagulated patients because of the risk of bleeding,^4^ which is the most common complication and occurs 1–2 weeks after the procedure in 2–7% of patients.^5,6^

**Table 1:** Goligher classification of haemorrhoids

Grade	Description
I	Haemorrhoids that bleed but do not prolapse
II	Haemorrhoids that prolapse but spontaneously reduce
III	Haemorrhoids that prolapse but have to be manually reduced
IV	Haemorrhoids that prolapse but cannot be reduced

Adapted from *Surgery of the Anus, Rectum and Colon*^3^

An alternative therapy for patients receiving anticoagulation is injection sclerotherapy (IST) with 5% oily phenol in almond oil, with a reported overall success rate of 93–98% in patients with grade I–III haemorrhoids.^7,8^ The IST causes a local reaction in the venous plexus of the haemorrhoid, permanently damaging the endothelium.^9^ In contrast to RBL, the reported rate of postprocedural bleeding is lower,^7^ suggesting that IST could be an alternative to RBL in the management of haemorrhoids in anticoagulated patients. However, although complications of IST are rare, infections of the urinary tract, including prostatitis and prostatic abscess, can occur when IST is not applied correctly into the haemorrhoidal cushions.^10^

In this study, we report the use of IST in patients with bleeding symptomatic haemorrhoids (SH), in whom anticoagulation is required, as they are at high risk of thromboembolic disease.

## Methods

This retrospective study was performed in a colorectal surgical unit, based alongside a tertiary cardiothoracic surgical service. Case notes and endoscopy records were searched to identify patients treated with an IST for SH between 1 April 2014 and 30 November 2021. The patients included were high-risk patients who were anticoagulated, had active bleeding from haemorrhoids, and in whom conservative treatment had failed. In these patients, anticoagulation therapy could not be stopped safely because of the risk of thrombosis or embolism from cardiac or valvular disease. Two colorectal surgeons performed the procedures using a standardised technique.

The primary outcome of the study was successful treatment of SH, defined as resolution of bleeding for six months. Following each IST treatment, patients were advised to contact the colorectal department if they had ongoing bleeding for a clinical review and further IST treatment if appropriate. If no contact had been received after six months, the treatment was considered successful. Case notes were also searched to establish whether the IST treatment had been successful when the patient was reviewed in the outpatient clinic. Where no contact had been received from the patient, or they were not reviewed in the outpatient clinic, patients were contacted by telephone to establish whether they were experiencing ongoing bleeding. Any reported bleeding after 6 months was considered a recurrence. Another procedure in the same patient was considered a procedure, not another patient episode. The secondary outcomes were patient-reported complications, number of IST procedures and number of other procedures performed to achieve symptom resolution.

### Injection sclerotherapy procedure

In all cases, the procedure was performed on a day-case basis in an endoscopy unit, or operating theatre if no capacity was available in the endoscopy suite. Patients provided informed consent highlighting the potential risks including the need for additional IST procedures, which was documented on a standardised consent form. The patient was placed in the left lateral position and examined using a proctoscope inserted into the anal canal with lubrication combined with local anaesthetic. The sclerosant used was 5% oily phenol (AAH Pharmaceuticals Ltd, UK), which was injected into the haemorrhoidal cushions above the dentate line using a haemorrhoid injection needle. The volume of the sclerosant used varied at the discretion of the surgeon. Following the procedure, the patient was observed in the endoscopy suite recovery room as would be routine following an endoscopy procedure performed without intravenous sedation, typically for about an hour.

## Results

A total of 20 patients (median age, 64 years [range 35–86], 14 male) who underwent 32 IST procedures between 1 April 2014 and 30 November 2021 were identified (Table 2). All patients had either symptomatic grade II (*n*=12, 60%) or III (*n*=8, 40%) haemorrhoids, and were anticoagulated with either single or combination drug therapy (Table 2). Only one patient was treated in an operating theatre because of reduced capacity in the endoscopy unit. In all cases, the patients were discharged on the same day after a brief period of postprocedure observation.

**Table 2:** Patient baseline demographic and clinical data

	Patients, *n*=20
Age, median (range)	64 (35–86)
Male/Female	14/6
Goligher classification, *n* (%)	
I	0 (0%)
II	12 (60%)
III	8 (40%)
IV	0 (0%)
Anticoagulation regime, *n* (%)	
Novel oral anticoagulant	10 (50%)
Apixaban	6 (30%)
Rivaroxaban	4 (20%)
Warfarin	6 (30%)
Dual Anticoagulation	4 (20%)
Clopidogrel and Warfarin	1 (5%)
Clopidogrel and Rivaroxaban	1 (5%)
Clopidogrel and low molecular weight heparin	1 (5%)
Aspirin and Ticagralor	1 (5%)
Anticoagulation indication, *n* (%)	
High-risk venous thromboembolic disease	10 (50%)
Recent cardiac surgery	3 (15%)
Atrial fibrillation	4 (20%)
Recent myocardial infarction	3 (15%)

In the included patients, 32 IST procedures were performed with a mean volume of sclerosant of 5.64ml (range 2.5–10ml).

**Figure 1:**
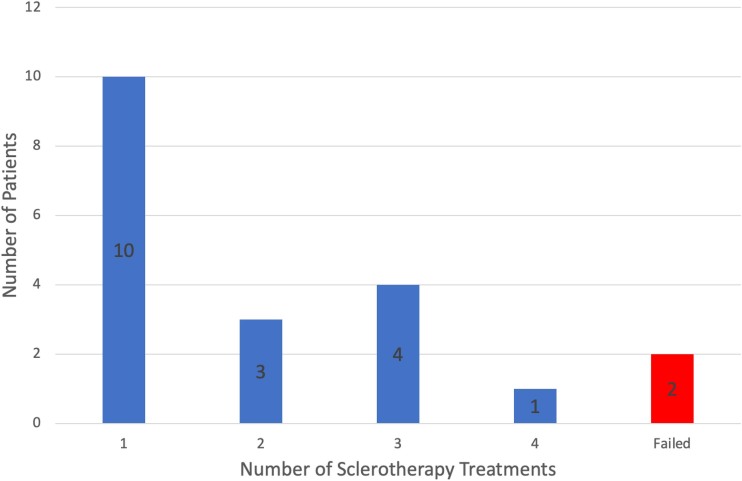
Number of injection sclerotherapy procedures required to achieve symptom resolution

In 18 patients, the IST treatment was performed while the patients remained on their established anticoagulation regimen. Symptom resolution was achieved in ten patients (50%) after a single IST procedure. Three patients (15%) showed resolution of symptoms after the two procedures. The remaining five patients required three (*n*=4, 20%) or four (*n*=1, 5%) interventions (Figure 1), representing an overall success rate of 90% using IST alone (*n*=18), with 65% (*n*=13) achieving symptom resolution after up to two IST treatments. In patients requiring more than one IST treatment, the median interval between the two procedures was 31.5 weeks (range 8–133 weeks).

Patients were reviewed in the outpatient clinic, or contacted by telephone, at a median of 7 months (range 1–27) following their last IST treatment. Fifteen (75%) patients reported no ongoing bleeding, whereas in two (10%) anticoagulation was suspended to enable further procedures. One patient who had already received one IST treatment was referred by his general practitioner to another colorectal unit and underwent successful RBL treatment. A further patient had received two IST treatments but suffered ongoing bleeding and progression of his haemorrhoid disease, ultimately requiring a haemorrhoidectomy. Three (15%) patients were not reviewed in an outpatient clinic and were not contactable at the time of this study.

No patient reported complications following IST treatment, and no complications were identified in the immediate postprocedure period while the patient remained in the hospital.

## Discussion

Several treatments are available for SH; however, in anticoagulated patients, a significant number of these procedures are contraindicated due to the potential risk of bleeding. Although anticoagulation can be temporarily and safely withheld in many cases, in a small number of patients at high risk of thromboembolic events,^11,12^ it can be unsafe to withhold anticoagulation to allow haemorrhoid treatment. In addition, suspending anticoagulation therapy can be challenging for patients and requires a period to elapse before the treatment can proceed. IST has the potential to control haemorrhoidal bleeding without the need to suspend anticoagulation in patients at risk of adverse thromboembolic events, or where rapid treatment is required. By not suspending anticoagulation therapy, patients and clinicians avoid the inconvenience of discontinuing and restarting medications at defined intervals. This study in our colorectal unit is one of the largest reported single-centre series of anticoagulated patients receiving IST for SH and demonstrates that it is a potentially safe and effective treatment option to control haemorrhoidal bleeding.

IST is an outpatient procedure that has been used effectively in other patient groups with SH. The learning curve and risk of complications if injected in the wrong plane are potential causes for concern.^10,13^ However, IST does not leave a raw surface in the anorectum, with the associated risk of secondary haemorrhage.^7^ Therefore, it may be an effective and safe treatment option for SH in high-risk anticoagulated patients. Various sclerosants, including 3% polidocanol and 5% oily phenol, are available. They have a similar mechanism of action by inducing fibrosis in the target tissue.^9^ Whereas IST is commonly used in grades I–II (internal) haemorrhoids,^14^ there is precedent for its use in symptomatic grade III haemorrhoids, as in this study, because of the perceived low risk of complications including postprocedural bleeding.^7,8,15^

In 2019, Fernandes *et al* evaluated the use of IST with 3% polidocanol foam in their study of 210 patients, which included 22 (10.5%) patients receiving concurrent anticoagulation.^8^ They concluded that IST was a safe and effective treatment for SH in anticoagulated patients; however, two patients receiving anticoagulation with dual agents experienced significant postprocedural bleeding requiring blood transfusion. In a similar study using 3% polidocanol foam, Salgueiro *et al* reported an overall success rate of 93.4% and bleeding in 5.5% (4/73) of patients with bleeding disorders or those receiving anticoagulants.^7^ Our study reported success in 65% (*n*=13) of patients receiving up to two IST procedures, with an overall success rate of 90% using IST alone while maintaining patients on anticoagulation therapy. Our study reported no postprocedural bleeding requiring intervention or patient-reported complications.

Although our study demonstrated that IST is a potential treatment option for anticoagulated patients, it does have several limitations. This study was retrospective in design, with a small sample size, and relied on patient-reported complications, with no further contact as a surrogate marker of symptom resolution. Further prospective studies with larger cohorts are required to establish the success and complication rates more reliably.

## Conclusion

Our study demonstrated that IST with 5% oily phenol is a potentially safe and effective procedure for treating SH in high-risk, anticoagulated patients. However, further work is required in a prospective study with a larger cohort before IST can be the established treatment of choice for these patients.

## Data access statement

Data supporting this study can be provided upon reasonable request.

## Ethics

This study had a retrospective case series design; therefore, ethical approval was not required.
